# Cocreation of a Mobile App (AYABytes) by Physicians and Adolescents and Young Adults With Cancer to Improve Access to Cancer-Related Resources and Reduce Distress: Protocol for a Single-Arm Feasibility Study

**DOI:** 10.2196/69453

**Published:** 2025-12-01

**Authors:** Evelyn Yi Ting Wong, Brian Shao Tian Woon, Wei Lin Goh, Sri Nur Qurniany Binte Abdul Latif, Nurulshazwani Bte Mohd Shahrudin, Victoria Hwei May Wong, Daniel Song Chiek Quah, Yee Pin Tan, Yung Ying Tan, Selene Tze Ling Chan, Eileen Yi Ling Poon, Mohamad Farid

**Affiliations:** 1 Division of Medical Oncology National Cancer Centre Singapore Singapore Singapore; 2 Division of Supportive and Palliative Care National Cancer Centre Singapore Singapore Singapore; 3 Department of Psychosocial Oncology National Cancer Centre Singapore Singapore Singapore; 4 Department of Nursing Singapore General Hospital Singapore Singapore

**Keywords:** adolescents and young adult oncology patients, mobile app, innovation, oncology, cancer

## Abstract

**Background:**

Adolescents and young adults with cancer require dedicated and tailored management that bridges adult and pediatric oncology services. At the National Cancer Centre Singapore, 40% of newly diagnosed adolescents and young adults report significant distress due to uncertainty about prognosis, treatment, and disruption of life milestones. A major unmet need is access to reliable, age-appropriate information. Prior studies demonstrate that digital technology can effectively deliver such support.

**Objective:**

This study describes the protocol for evaluating AYABytes (Adolescent and Young Adult Building Youths, a Technology for Education and Sharing), a mobile app cocreated by patients and health care professionals to improve health-related quality of life for adolescent and young adult oncology patients. An iterative information-gathering process was conducted, including semistructured interviews with 2 clinicians, 3 cancer survivors, and 3 care partners to cocreate this mobile app. AYABytes is an interactive, phone-based intervention designed to engage adolescent and young adult oncology patients with personalized education, mood, and symptom self-management resources with an inbuilt algorithm that responds to patient-reported questionnaires.

**Methods:**

The app will be evaluated in 2 phases—a pilot test and an implementation test. In the pilot test, the app will be launched to a test group of 20 adolescent and young adult oncology patients aged between 16 and 45 years, selected for representation among the age group and their malignancies. Patients will be allowed to use the app for 1 month. Feasibility and acceptability were assessed via a semistructured survey. In the implementation stage, 200 patients will be allowed to use the app over 6 months and will complete an EQ-5D-5L questionnaire at baseline and at the 1- and 6-month marks. Evaluation of the mobile app was performed via the mHealth App Usability Questionnaire at similar intervals.

**Results:**

Funding for the development and trial of AYABytes was awarded in October 2020 through a National Cancer Centre Singapore research grant. Pilot testing was completed in May 2024. The implementation phase began in June 2024 and is currently ongoing.

**Conclusions:**

We believe that AYABytes, a novel eHealth mobile app, will be both beneficial and easily used by adolescent and young adult oncology patients. Evaluating the app and its quantifiable impact on improving the quality of life of adolescent and young adult oncology patients will help enrich the evidence for mobile health interventions. It will also validate new digital approaches to help adolescent and young adult oncology patients reduce their distress and address unmet needs and concerns.

**International Registered Report Identifier (IRRID):**

DERR1-10.2196/69453

## Introduction

### Background

Adolescent and young adult oncology patients are a unique group of individuals who are starkly different from their pediatric and adult counterparts. Defined as patients with cancer diagnosed between the ages of 16 and 45 years, they face significant medical and psychosocial challenges during and after treatment [1].

A recently published pilot study in our institution found that up to 40% of our adolescents and young adults with cancer have a significant distress level in the first 6 months of diagnosis [2]. Much of these distresses are related to a lack of age-appropriate information, a perceived lack of knowledge with regard to treatment details, possible side effects, financial concerns, and generalized anxiety. Studies and reports have shown that a large proportion of adolescent and young adult oncology patients are dissatisfied with the amount and quality of information and education that they receive during and after their cancer journey [3-5]. This lack of information also results in a negative impact on cancer control [6]. In addition, it has been found that up to 50% of adolescent and young adult oncology survivors report having their information and service needs unmet. This percentage of unmet information needs was similar regardless of cancer type [3] and despite the fact that many adolescent and young adult oncology patients report that receiving such information is important to them [7].

The methods by which people obtain information have been largely revolutionized with the advent of the internet and mobile gadgets. Digital health interventions (DHIs) offer a much-needed solution to plug this information gap in an accessible manner. Digital health platforms can be used in multiple ways, including for information delivery, 2-way communication, or longitudinal assessment [8]. The widespread use of the internet for health purposes has enhanced the ease of accessibility of DHIs [9]. This is particularly relevant for adolescents and young adults, who are pervasive users of technology.

Over the years, there has been a surge in the number of studies investigating the potential benefits of DHIs. However, adolescent and young adult oncology patients remain excluded from many of these important trials. A scoping review conducted by Lee et al [10] showed that the most common population involved were adults and older adults, with cancer survivors being the primary recipients of DHIs across studies. In addition, most DHIs target patients with early-stage cancer or patients with cancer in general. The benefit of DHIs should not be limited to cancer survivors; it should also be extended to adolescent and young adult oncology patients while they navigate their cancer journey. Adolescent and young adult oncology patients receiving treatment must make difficult decisions that can affect their long-term health and well-being [11]. Due to the often intense treatments that they have to undergo, many experience severe side effects during treatment. They also have to deal with long-term treatment-related toxicities and face a higher risk of secondary cancers during survivorship. DHIs that offer self-managing services and patient education regarding red flag symptoms will certainly improve outcomes of adolescent and young adult oncology patients [9,12,13]. AYABytes (Adolescent and Young Adult Building Youths, a Technology for Education and Sharing) hopes to be able to address this need by being a 1-stop platform where adolescent and young adult oncology patients, regardless of which stage of the cancer journey they are at, can obtain the necessary information that they need.

### This Protocol

This paper aims to describe the protocol for the development and evaluation of a digital app, AYABytes, that provides rapid access to information relevant to the cancer journey of Asian adolescent and young adult oncology patients. This will allow our own and regional adolescent and young adult oncology patients to have an accurate resource that they can turn to. By reducing barriers to information access, AYABytes will bring information sharing to a new level and pave the way for digital analytics in the future.

## Methods

### Overview

This is a single-center interventional study with the primary aim of developing a digital app to provide rapid access to information relevant to the cancer journey of adolescent and young adult oncology patients and to evaluate the capacity to implement this as an intervention arm.

We hypothesize that adolescent and young adult oncology patients prefer a digital informative platform in the form of an app to assess oncology-related educational materials. Our primary aim is to develop a digital app designed to provide rapid access to information relevant to their cancer journey and to evaluate the feasibility and acceptability of this digital app. We hypothesize that this app will be used actively by 60% or more of adolescent and young adult oncology patients. Feasibility will be determined by the percentage of participants who log into the app at least 2 times within the 6 months. We will assess the acceptability of the app with the mHealth App Usability Questionnaire (MAUQ) as our objective measure of satisfaction at the 1- and 6-month mark of use. We will be administering questionnaires to evaluate their preferences and their adoption as well. Frequent users are determined as 2 log-ins into the mobile app within 6 months, while nonfrequent users are determined as less than 2 log-ins into the app within the 6-month study period.

Our secondary aim is to measure the improvement in distress levels (National Comprehensive Cancer Network [NCCN] distress thermometer [DT]) for the frequent users of the app for further efficacy studies and the 5-level EQ-5D to study changes in health-related quality of life among young adults with cancer after using the app. We will measure distress levels and the EQ-5D before using the app, and at the 1- and 6-month marks after using the app.

### Participation and Recruitment

This study aims to recruit 200 newly diagnosed adolescent and young adult oncology patients over 18 months from the National Cancer Centre Singapore (NCCS) clinics in the Outram Campus. Eligible patients will be identified by their attending physicians and consented to during their in-person clinic consultation.

For this study, we will include patients who (1) are Singaporean citizens or permanent residents, (2) are aged between 16 and 45 years (parental consent will be obtained for patients aged <21 years), (3) speak English, (4) are newly diagnosed with any malignancies, (5) have the ability to provide informed consent and adequate mental capacity for the completion of the questionnaire, and (6) have brain metastases or primary brain cancers as long as they can provide informed consent and have adequate mental capacity for completion of the questionnaire. We will exclude patients who (1) are unable to comply with the follow-up and study protocol and (2) have medical conditions deemed inappropriate by the primary oncologist.

### Development and Data Sharing

The primary intervention is a smartphone app, AYABytes, which is a mobile health app designed for adolescent and young adults with cancer and cancer survivors. After the initial literature review, a team for idea generation was set up, consisting of 3 adolescent and young adult oncology patients, 1 medical oncologist, 1 palliative care physician, 1 medical social worker, 1 nurse, and 2 research associates. A cocreation project was subsequently embarked on to develop and design a smartphone app for adolescent and young adult oncology patients.

To inform the development and design of the AYABytes app, a targeted literature search was conducted between October 2020 and March 2021 using PubMed, Scopus, and Google Scholar. Search terms included combinations of “adolescent and young adult oncology,” “digital health interventions,” “mobile application,” “patient education,” “co-design,” “health literacy,” and “psychosocial support.” Studies were included if they were published in English within the past 10 years and focused on digital interventions for adolescents and young adults with cancer or other chronic illnesses. Gray literature, institutional reports (eg, the World Health Organization and National Cancer Institute), and app store reviews were also examined to benchmark app features, usability design principles, and engagement strategies. This process informed the selection of core functions, such as symptom tracking, personalized educational content, and storytelling, and guided the choice of validated evaluation instruments such as the MAUQ.

In parallel, we conducted a review of digital health implementation frameworks and outcome measures, focusing on feasibility and usability assessment tools. We also performed a legal and regulatory review of local and international data protection standards, including the Singapore Personal Data Protection Act and the General Data Protection Regulation, to guide data governance and risk management planning. Security clearance for the app was obtained from Synapxe, Singapore’s national HealthTech [14] agency, to ensure infrastructure compatibility, cybersecurity compliance, and adherence to institutional standards for mobile health app deployment.

The study mobile app will be an added tool for the participants’ cancer care delivery process. The app will be developed by an independent mobile app developer, and licensing will be provided to SingHealth Synapxe. Participants will be logged into the app via an anonymous and deidentified username. No patient details will be obtained through the mobile app. We intend to design and build this app by working with a specialized app developer, Stone&Archer. The technology selected for implementing the intervention is a smartphone app (for both the Android and Apple iOS systems). Curated and appropriate information will be uploaded into this app. There will be inbuilt algorithms that the patient can select to further individualize the information received to personalize their experience with the interface.

### Primary Intervention and AYABytes App Features

Information sharing is an important mediator of patient outcomes [14]. From our pilot study, we have identified the key concerns that an adolescent and young adult oncology patient may have in the local Singapore context. These concerns fall into the following domains: (1) information about cancer diagnosis, (2) investigation process and implications, (3) treatment process and possible complications, (4) fertility preservation, (5) financial costs, (6) cancer screening and prevention, and (7) diet and complementary medicine. Consequently, AYABytes will contain substantial informative articles, endorsed by NCCS oncologists, covering the abovementioned topics. AYABytes seeks to reduce the distress experienced by adolescent and young adult oncology patients by resolving the uncertainty that comes with insufficient understanding surrounding their diagnosis, and current and long-term plans.

**Figure 1:**
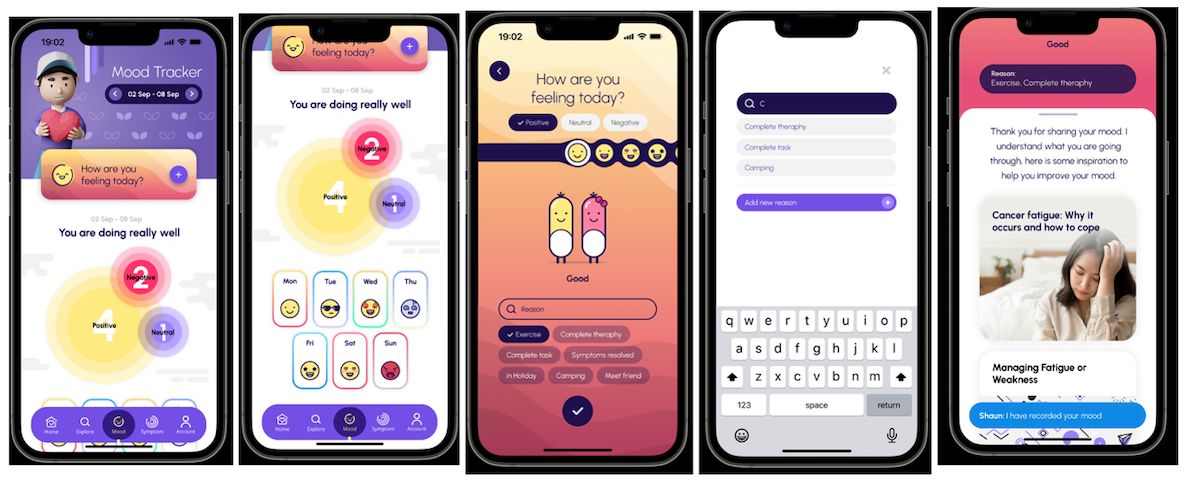
Mood and symptom tracking interface.

The AYABytes framework is anchored on 3 core design tenets—information sharing, individualized care, and community support—each linked to specific in-app features (Table 1). AYABytes hopes to achieve this by giving adolescent and young adult oncology patients a platform to chart the physical manifestations of their cancer, the side effects of their treatment, and their emotional response toward it in real time (Figure 1). With this visual overview of how the cancer treatment affects the adolescent and young adult oncology patients’ life, the team can optimize the treatment regimen such that their well-being is not significantly compromised in favor of inconsequential gains in treatment efficacy. Nonetheless, this monitoring of patient-reported outcomes also helps adolescent and young adult oncology patients better manage their symptoms, increase satisfaction with their treatment, and improve communication with their physicians regarding their concerns and expectations [15].

**Table 1:** Overview of the design tenets underpinning the construct of AYABytes (Adolescent and Young Adult Building Youths a Technology for Education and Sharing).

Design tenets	Associated key AYABytes features
Information sharing	Informative articles endorsed by National Cancer Centre Singapore oncologists on topics relevant to local adolescent and young adult oncology patients
Individualized care	In-app mood and symptom tracking
Community support	Testimonials by fellow local adolescent and young adult oncology patients

**Figure 2:**
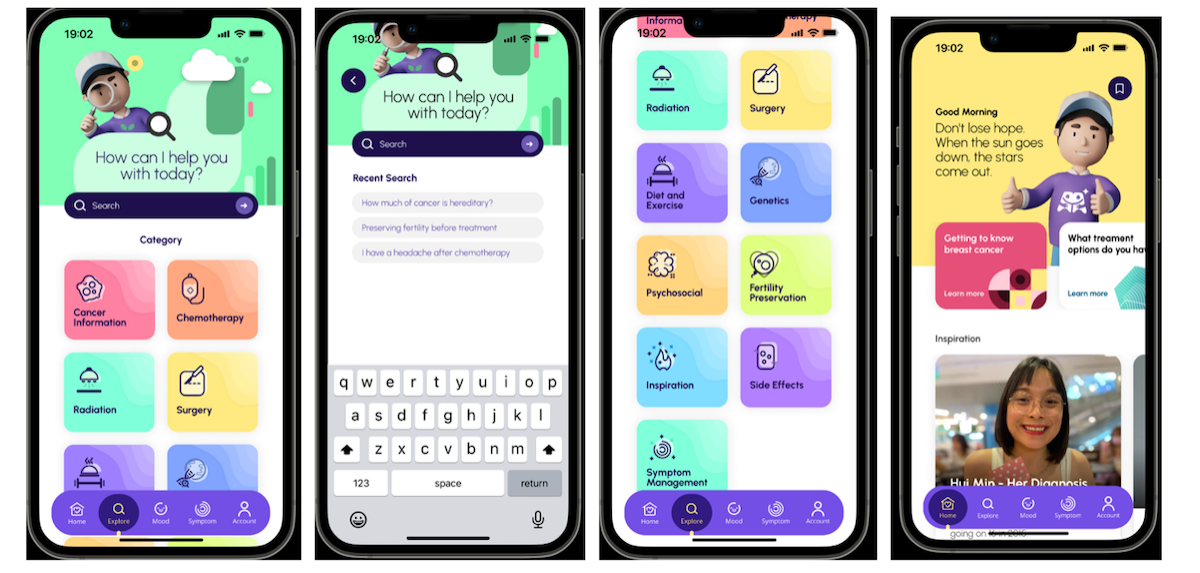
Cancer-specific articles and testimonies of cancer survivors.

AYABytes’ last tenet focuses on the creation of a supportive community for adolescent and young adult oncology patients to find peace and solace when it comes to coping with their disease. Receiving a cancer diagnosis is destabilizing, and many adolescent and young adult oncology patients experience an increase in distress in trying to maintain a normal lifestyle while coping with cancer. Furthermore, it is not uncommon for adolescent and young adult oncology patients to feel alone throughout the course of their cancer journey [16]. AYABytes will contain authentic patient-submitted stories of their journey through their cancer diagnosis (Figure 2). Reading these first-hand patient narratives allows other adolescent and young adult oncology patients to have a better idea of what they are about to go through. More importantly, it shows them that their experience is not unique, that many before them have had a similar experience, and that they will be able to find the strength to make it through this difficult period as well [17].

### Phase 1: Pilot Testing

The app will be launched to a test group of 20 adolescent and young adult oncology patients. Recruitment will be targeted for an appropriate representation of gender and age groups across the adolescent and young adult oncology patient spectrum. During the pilot test, patients will use the app over the course of 4 weeks. During both phases 1 and 2, patients will not be given any specific instructions on the frequency with which they should use the app. They will be instructed to use the app as they see fit. Patients will provide their evaluation on the feasibility and acceptability of the app through a semistructured survey.

### Phase 2: Implementation Testing and Quality of Life Evaluation

There are various outcome assessments that are part of this study. The primary aim would be to evaluate AYABytes’ feasibility as well as usability among adolescent and young adult oncology patients. Secondary end points would include the estimates for the extent of improvement in distress levels (NCCN DT) as well as health-related quality of life (EQ-5D-5L) for the frequent users of the app. Table 2 shows the surveys that will be administered to the participants during the study.

**Table 2:** Survey assessment timeline for recruited adolescent and young adult oncology patients.

Time	Surveys
Baseline	NCCN^a^ distress thermometer EQ-5D-5L
First month	MAUQ^b^ NCCN distress thermometer EQ-5D-5L
Sixth month	MAUQ NCCN distress thermometer EQ-5D-5L Exit interview survey

^a^NCCN: National Comprehensive Cancer Network.

^b^MAUQ: mHealth App Usability Questionnaire.

Feasibility is determined based on how frequently adolescent and young adult oncology patients use AYABytes. Frequent users are determined as having at least 2 log-ins into AYABytes within the 6 months, while nonfrequent users are determined as having less than 2 log-ins into AYABytes over 6 months.

Usability is assessed via a few metrics. We use the MAUQ as our objective measure of satisfaction. The MAUQ was designed by Zhou et al [18] based on several existing questionnaires used in previous mobile app usability studies. Psychometric analysis indicated that the internal consistency of MAUQ is high and correlates strongly with many well-validated questionnaires (System Usability Study and Poststudy System Usability Questionnaire). The MAUQ will be administered at the 1- and 6-month mark.

Usability will also be assessed via the following objective outcome measures: (1) the proportion of eligible participants enrolled and reasons for nonparticipation, (2) proportion of participants retained in the study, and (3) intervention fidelity (ie, occurrence of technical problem). The number of individuals assessed for study eligibility, reasons for exclusion or noncompletion, and numbers included in the analysis will all be recorded to examine the acceptance of the intervention and identify issues with the intervention or technology affecting participation.

All participants will undergo a final exit survey within 1 month after completion of the study. The exit survey will involve feedback regarding the impact of the intervention, its utility, relevance of the algorithms, value or burden of item repetition in the algorithm, symptoms or problems that were not addressed by the mobile app, and general comments. Participants will also be asked to share what actions have been taken in response to the information obtained from AYABytes.

We intend to use the NCCN DT as the objective measure of distress level. This was chosen due to its ease of use and specificity. It has also been validated for use in patients with cancer in Singapore and has been used in studies concerning local adolescent and young adult oncology patients [19]. The DT involves the following five domains: (1) practical, (2) family, (3) emotional, (4) spiritual and religious, and (5) physical problems. The DT functions on an ordinal scale from 0 to 10, with 0 being the lowest and 10 being the highest score. A score of more than 4 would correspond to clinically significant distress in patients with cancer according to current guidelines.

**Figure 3:**
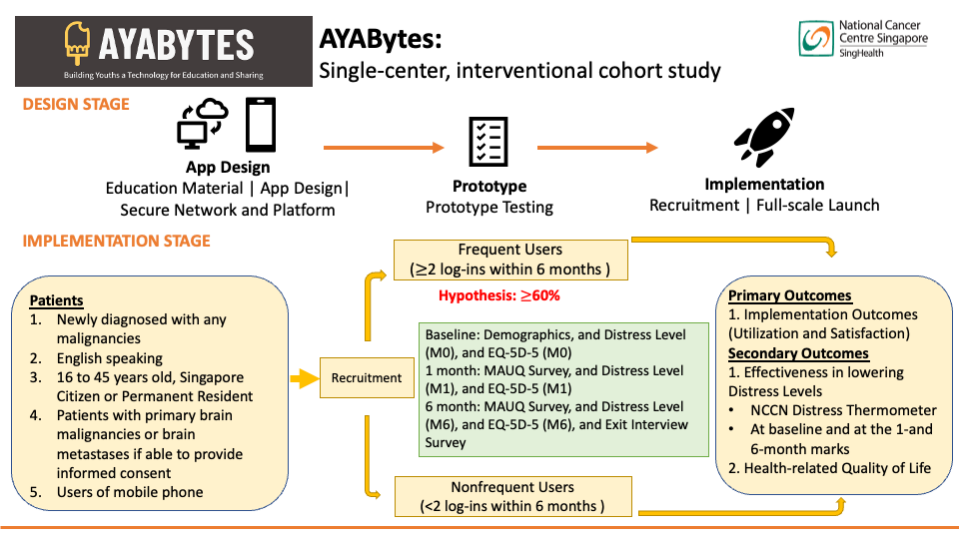
Overview of the AYABytes (Adolescent and Young Adult Building Youths, a Technology for Education and Sharing) protocol.

In this study, we will also pilot the use of the 5-level EQ-ED (EQ-5D-5L) questionnaire for future efficacy studies [20]. This questionnaire produces a 5-digit health state profile representing the level of reported problems on each of the 5 dimensions of health. They include mobility, self-care, usual activities, pain or discomfort, and anxiety or depression. Therefore, we have chosen to pilot the EQ-5D-5L because of its widespread use and validated multiattribute utility for measuring health-related quality of life. An outline of the study protocol is depicted in Figure 3.

### Statistical Analysis

The primary end point of this study is the proportion of frequent users of the digital app. Assuming the proportion of frequent users to be 50%, a sample size of 97 patients is required to estimate this proportion with a 2-sided 95% CI and a 10% margin of error. We have assumed 50% of the users to be frequent users because this will require the largest sample size to achieve the specified precision of the 95% CI for the parameter of interest. In the pilot study, 60% of the patients completed all the DT questionnaires at baseline, 1 month, and 6 months. Adjusting for a 40% dropout rate, 160 patients will be recruited in this study.

All data collected in this study will be summarized using descriptive statistics. Frequency and percentages will be reported for categorical variables. Median with interquartile ranges or mean with SD will be reported for continuous variables. The proportion of frequent users will be reported with a 95% CI estimated using the exact method. Distress levels will be reported as a proportion of adolescent and young adult oncology patients with a score of greater than 4 for each of the 39 items in the DT at baseline, 1 month, and 6 months. Mean and SD of the total distress score will also be reported across the 3 time points, together with any changes in total distress score at 1 and 6 months from baseline. Satisfaction ratings will be reported for 1 and 6 months. Results on distress levels, satisfaction ratings, and exit surveys will be broken down by frequent and infrequent users of the app.

### Ethical Considerations

All research involving human participants is reviewed and approved by the SingHealth Institutional Review Board (2022/2559). Participants will provide informed consent after receiving detailed explanations of the study’s objectives and procedures. Participant privacy and confidentiality were rigorously maintained. Identifying information will be anonymized or omitted in reporting, and the data will be stored securely in compliance with institutional and legal standards. No compensation will be provided.

## Results

Funding for the development and trial of AYABytes was awarded in October 2020 through a NCCS research grant. The design team conducted 3 cocreation meetings with adolescents and young adults, clinicians, and care partners between October 2020 and June 2021 to inform the app’s initial features and content. The design and programming of the AYABytes mobile app for both Android and iOS platforms were completed in June 2023. The app is optimized for compatibility across both operating systems.

Pilot testing of AYABytes was completed in May 2024 and involved 20 adolescent and young adult participants. The implementation phase began in June 2024 and is currently ongoing. Recruitment for the implementation phase is expected to conclude by December 2025. An overview of the study design, key phases, participant activities, and timeline is presented in Table 3.

**Table 3:** Overview of study design, phases, participant activities, and timeline for the AYABytes (Adolescent and Young Adult Building Youths a Technology for Education and Sharing) protocol.

Phase	Activity	Sample size	Duration	Outcomes assessed
Predevelopment (October 2020-June 2021)	Literature review, stakeholder interviews, legal and cybersecurity review, and app prototype development	—^a^	9 months	App design requirements and privacy compliance
Development phase (July 2021-June 2023)	App design, programming, and internal testing with developers	—	24 months	Functionality verification and internal quality assurance
Phase 1: pilot study (March-May 2024)	Pilot-testing of the app among 20 adolescent and young adult oncology patients	20	4 weeks per participant	Feasibility, acceptability, and MAUQ^b^ usability
Phase 2: implementation study (June 2024-December 2025)	Full rollout to 200 participants	200	6 months per participant	EQ-5D-5L, NCCN^c^ distress thermometer, and MAUQ

^a^Not applicable.

^b^MAUQ: mHealth App Usability Questionnaire.

^c^NCCN: National Comprehensive Cancer Network.

## Discussion

### Anticipated Findings

The landscape of cancer management in the current age is very different from that just 10 years ago. Patients, especially adolescent and young adult oncology patients, are taking more control over their health. It has become commonplace for patients to search for their symptoms from multiple online sources before their first consultation with a physician. This dependence on online services as the first-line management for the occasional benign ailment certainly does not cause much harm [21]. However, this may not be as reliable when it comes to seeking out cancer information as cancer care is often more complex. With uncurated information on the internet, any unverified information may potentially be nebulous at best [22]. This uncertainty can lead to more significant distress regarding their symptoms and disease. Moreover, sifting through tons of conflicting data often leads to delays in accessing health care services and its associated medical complications [23,24].

This shift is paralleled by a corresponding change in patient expectations within the health care system, moving from the traditional paternalistic approach to a more collaborative model [25]. Patients want to participate actively in their care and desire transparency and shared decision-making [26]. In this fast-paced health care environment, patients increasingly expect swift access to health information and solutions to their concerns [21]. With abundant health information online, they are driven to conduct their research and arrive at appointments with established ideas and concerns [22]. This underscores the importance of creating reliable health resources for patients to access as needed, as their primary team cannot realistically address their concerns with the same efficiency as the various online platforms. DHIs allow for patients’ concerns to be addressed proactively, promptly, and accurately while concurrently optimizing the efficiency and effectiveness of their next face-to-face consultation. DHIs have demonstrated their effectiveness in improving health care outcomes, but it is crucial to recognize that they cannot function in silos. DHIs improve patients’ quality of life and functional capacity while reducing the frequency of unplanned admissions and the total duration of hospitalization [27,28]. However, they do not replace regular care or contact time with physicians. Thus, while DHIs hold immense promise, their true potential lies in their integration and synergy within broader health care systems.

With the current diversity of DHIs available on the market, it is vital to take a step back to identify critical features of existing DHIs that allow them to have a long-standing impact on adolescent and young adult oncology patients. DHIs need to be intuitive, engaging, and user-friendly. It also needs to be comprehensive and safe [10]. With this understanding, AYABytes will be prototyped and undergo consumer testing before final implementation. All information available on the app will also be evaluated for clarity and accuracy. Perhaps more prominently, the next frontier for DHIs, such as AYABytes, that seek to offer care throughout the cancer continuum would be connectivity and systems integration. We aim for AYABytes to be linked with local health care systems so that adolescent and young adult oncology patients outside the NCCS and the broader SingHealth infrastructure will benefit from it. Consequently, this will also allow for more convenient follow-up and management of adolescent and young adult oncology patients throughout Singapore. With all these strategies laid out, we have optimistic expectations for AYABytes to catalyze the transformation of cancer care delivery for local adolescent and young adult oncology patients and improve their cancer outcomes, ushering in a new era of cancer care.

### Limitations

This project could be limited by the population size of adolescent and young adult oncology patients in Singapore. We estimate the dropout rate to be 40%, requiring us to enroll at least 160 patients so that enough completed questionnaires will be available for analysis. With approximately 400 to 500 adolescent and young adult oncology patients diagnosed yearly, recruiting and retaining approximately 200 patients might pose a challenge. To mitigate and minimize this limitation, our study coordinator will follow up by calling patients who did not complete the online questionnaires to assist with collecting responses. We will aim to publish our final results once we have at least 97 patients completing all 6 months of survey assessments. In addition, given that AYABytes is a mobile app, we can only recruit patients who own a digital device running the Android or iOS system. Given the age range of our sample population, there will likely be minimal selection bias.

### Conclusions

AYABytes represents a contemporary approach to digital health. Its function as an encyclopedia of adolescent and young adult oncology patient–specific cancer information will improve health literacy, patient engagement, and health outcomes among adolescent and young adult oncology patients. With additional features, such as symptom monitoring and sharing of patient stories, AYABytes seeks to capture the essence of what it means to be diagnosed with cancer in Singapore in this decade. Adolescent and young adult oncology patients are not alone in their recovery, and AYABytes represents this collaboration between the physician and the patient, with the wider adolescent and young adult oncology patient community playing a crucial role. This protocol paper describes the process of creating and executing the AYABytes app. With the positive feedback and interest gathered from our pilot study, we are optimistic that AYABytes will live up to the expectations of our adolescent and young adult oncology patients as a reliable cancer resource for years to come, with the entire adolescent and young adult oncology patient community serving as active participants.

## Appendix

Multimedia Appendix 1 Peer review comments from the Institutional Fund Committee, National Cancer Centre Singapore.

## Abbreviations

AYABytes: Adolescent and Young Adult Building Youths, a Technology for Education and Sharing
DHI: digital health intervention
DT: distress thermometer
MAUQ: mHealth App Usability Questionnaire
NCCN: National Comprehensive Cancer Network
NCCS: National Cancer Centre Singapore
