# Supplementary material for: Cocreation of a Mobile App (AYABytes) by Physicians and Adolescents and Young Adults With Cancer to Improve Access to Cancer-Related Resources and Reduce Distress: Protocol for a Single-Arm Feasibility Study
Source: JMIR Res Protoc. 2025 Dec 1;14:e69453. doi: 10.2196/69453 (PMC12706439; doi:10.2196/69453)
Supplement: Multimedia Appendix 1 [file resprot_v14i1e69453_app1.pdf]

National Cancer Centre Singapore
SingHealth

NCCS CANCER FUND
Part of SingHealth Fund

Doc. No.:	DCOP-GA-GM-001
Appendix:	6G
Version:	1.5
Date:	1 December 2019

GRANT REBUTTAL (RESEARCH)

Please complete the form below in addressing the reviewer's comment and return in soft copy to NCCS Cancer Fund (Research) c/o Department of Governance and Administration.
[redacted]

**Principal Investigator**	Dr. Wong Yi Ting Evelyn
**Category of Grant Application**	NCCS/ Division of Medical Oncology
**Title Of Research**	Digital Health Interventions for AYAOs: Development of a Mobile Application (AYABytes) to Improve Access to Cancer-related Information and Resources and Reduce Distress
**Amount requested**	$50,000

**For Official Use Only**	
Date Received by Governance & Administration	

In not more than 3 pages, please address the reviewers' major comments in the various areas.

Dear Reviewers,
Thank you for your time for your comments and thank you for considering this project.
Here are my replies to the comments for my NCCRF grant.

1) Approach/Methods: iHIS
iHIS takes care of the IT security across SingHealth institutions. We choose to work early in the project with iHIS in order to house the licensing of the app at the end of the development. In addition, while it is an app that is developed by an independent company, it is still a patient-facing application and hence there is a need to ensure user security. iHIS will also act as a mediator to ensure good security protocols are in placed during the development of the app for patients to use. As we are eventually planning collect research data as well from our patients who use the app, either through the app or in person, this digital security would be important.

2) Approach/Methods: Technical Preparation
To build an app, we will first need to understand coding. A basic app can be built using simple coding however we are keen to build an interactive app that will not only be used as a platform for information but can also be tailored according to the needs of a user. During our literature review, we had shortlisted two potential app development companies: Stone&Archer and

BYWORD. Both companies have worked with SingHealth before and have even developed MobileApps for SingHealth (eg.iECG by National Heart Centre was created by Stone&Archer in collaboration with iHIS.

The mobile app will house curated information in an intuitive manner however in order to make it customizable and tailored according to the needs of the user, we will need to use mobile app machine learning algorithms in order to have predictive analytic engines. In order to develop an app, we will first need to come up with the flow and specific features for the app. Secondly, we will need to design an overall look for the app. Thirdly, using a simple coding system like Xcode or Swift, the app will be constructed alongside the interaction design and flow template. Lastly, it will be submitted towards both the Android and iOS platform for use on both system. The data will also need to be housed on a secure cloud system like Amazon Cloud. For this, we will enquire the professional help from app development companies.

3) Approach/Methods: Potential App Development Companies
While researching on the different types of mobile app, we also considered the idea of a chatbot. We are currently under discussion with Bot.MD as well. Bot.MD can integrate with scheduling systems, storage drives, chemotherapy protocols, fertility protocols as well as Singhealth Academia information if necessary and will allow for fast integration. However we are still in discussion with them and will be pending an official quotation before we decide if the NCC Cancer Fund is sufficient to cover it.

4) Approach/Methods: Alternative – Cost effective/feasible
We had considered other cost-effective options including collaboration with students from National Technology University and other independent individuals however due to the sensitivity of a patient-facing app and our desire to eventually develop the app to allow integration with our clinical services, we decided to employ a professional company instead.

5) Principal Investigator and Technical Abilities
Dr Evelyn Wong Yi Ting is currently a Senior Resident in Medical Oncology however she has a strong interest in Digital Informatics. During COVID-19, she coordinated the design and implementation of a virtual combined Thoracic Tumor Board to discuss the clinical management of patient with Thoracic, Thymic and Pleural malignancies. She is also a member of the Medical Oncology Telehealth and Teleconsultation workgroup.

During her junior residency, she was a Director a Non-profit organization, named Seeds of Nation (SON Ltd) that collaborated with local medical community organizing medical camps in the rural villages of India, Himalayans and Thailand. The company organizes overseas community service trips to various developing nations, focusing on projects that bring health and education to the needy. Over the years, she has collaborated with the local communities there learning about telemedicine in the rural villages.

She has a keen interest in Digital Informatics and is keen to build her experience in Health Economics and Digital Informatics in the future.

Principal Investigator: Dr. Wong Yi Ting Evelyn

Signature: [signature]

Date: 6/12/20

Please email completed form before **7 Dec 2020, Monday** to NCCS Cancer Fund (Research) c/o [redacted].

National Cancer Centre Singapore
SingHealth

NCCS CANCER FUND
Part of SingHealth Fund

Doc. No.: DCOP-GA-GM-001
Appendix: 6G
Version: 1.5
Date: 1 December 2019

GRANT REBUTTAL (RESEARCH) R2

Please complete the form below in addressing the reviewer's comment and return in soft copy to NCCS Cancer Fund (Research) c/o Department of Governance and Administration. [redacted]

**Principal Investigator**	Dr. Wong Yi Ting Evelyn
**Category of Grant Application**	NCCS/ Division of Medical Oncology
**Title Of Research**	Digital Health Interventions for AYAOs: Development of a Mobile Application (AYABytes) to Improve Access to Cancer-related Information and Resources and Reduce Distress
**Amount requested**	$50,000

**For Official Use Only**	
Date Received by Governance & Administration	

In not more than 3 pages, please address the reviewers' major comments in the various areas.

Dear Reviewer,
Thank you very much for your time and input. Kindly refer to my answers to the comments below.

1. **In terms of the information – unclear how information will be invidualised and made age-appropriate. Authors mentioned customizable variables – age, gender, tumour type, married, single, education level, nationality but these are fairly broad categories and difficult to make information truly relevant to the individual.**

   Part of the reason why we decided to use these broad categories is mainly to test the function of individualized information within the app itself. As we will be developing the app from scratch, we intend to draw our own variables that we can portray individualized information as a testing ground. With regards to married, we will then offer the educational sites regarding fertility and likewise, depending on gender, we will then tailor it according to male vs female.

2. **What is the plan for the roll out? Simultaneously across all sites? Or targeting specific tumour types first? If so which tumour types.**

To clarify, we intend to first roll out and recruit patients only within National Cancer Centre Singapore – Outram Campus in order to recruit them for our study as well. We intend to allow all patients with newly diagnosed tumor types to use the app. Depending on the construction of the app, we will subsequently think of the roll out in phases. As we wish to include patients of all tumor type (breast, gynae, head and neck, lung, genitourinary, upper gastrointestinal, hepatobiliary, colorectal, brain, lymphoma and sarcoma), we will likely recruit all in increasing numbers, starting from 5 per tumor types and increasing recruitment numbers with time.

3. **Will there be a built-in option to allow patients to consent to information being released to their respective physician?**

   For this current version of the app, we do not intend to release these information to their respective physician as the app is currently mainly to push information out to the patient and not to collect quantitative or qualitative data of the patient through the app. Any data collected will be in person and through the data collection forms in person. In future, we are looking towards coming up with an app that will allow two way transfer of data as well.

4. **What is the long term maintenance/ tech support plan for the app once funding is exhausted?**

   We are hoping to include this digital integration as part of our clinical line and hoping to include this app as part of our SingHealth app like HealthBuddy. This will be pitched in the future once we have this preliminary data of feasibility.

5. **Questionaires are administered in the outpatient setting to obtain user feedback. Are there options for 'live feedback' during browsing as well? Will it be possible to build in interventional studies into the app – for example, testing different types of information along with readouts on patient satisfaction to guide adaptation followed by re-testing/ validation**

   Thank you for this question. It is possible to build in interventional studies into the app however this would means that it will require a secure cloud system to store data and information that we obtain from patients. Currently the best app for that would be HealthBuddy and the collection of information can be done via a data collection secure platform like ZDoc. This would likely have to be discussed in the future depending on the preliminary data and feasibility of this study.

Principal Investigator: Dr. Wong Yi Ting Evelyn

Signature: [signature]

Date: 15/12/20

Please email completed form before <u>**16 Dec 2020, Thursday**</u> to NCCS Cancer Fund (Research) c/o [redacted]
